# Identification of patients at risk for early death after conventional chemotherapy in solid tumours and lymphomas

**DOI:** 10.1054/bjoc.2001.2011

**Published:** 2001-09

**Authors:** I Ray-Coquard, H Ghesquière, T Bachelot, C Borg, P Biron, C Sebban, A LeCesne, F Chauvin, J-Y Blay

**Affiliations:** Centre Léon Bérard, Lyon, France; Institut Gustave Roussy, Paris, France; Hospital Edouard Herriot, Lyon, France

**Keywords:** lymphopenia, treatment-related death, cancer, risk factors, chemotherapy, cancer, palliative

## Abstract

1–5% of cancer patients treated with cytotoxic chemotherapy die within a month after the administration of chemotherapy. Risk factors for these early deaths (ED) are not well known. The purpose of this study was to establish a risk model for ED after chemotherapy applicable to all tumour types. The model was delineated in a series of 1051 cancer patients receiving a first course of chemotherapy in the Department of Medicine of the Centre Léon Bérard (CLB) in 1996 (CLB-1996 cohort), and then validated in a series of patients treated in the same department in 1997 (CLB-1997), in a prospective cohort of patients with aggressive non-Hodgkin's lymphoma (NHL) (CLB-NHL), and in a prospective cohort of patients with metastatic breast cancer (MBC series) receiving first-line chemotherapy. In the CLB-1996 series, 43 patients (4.1%) experienced early. In univariate analysis, age > 60, PS > 1, lymphocyte (ly) count ≤ 700 μl^−1^ immediately prior to chemotherapy (d1), d1-platelet count ≤ 150 Gl^−1^, and the type of chemotherapy were significantly correlated to the risk of early death (*P* ≤ 0.01). Using logistic regression, PS > 1 (hazard ratio 3.9 (95% Cl 2.0–7.5)) and d1-ly count ≤ 700 μl^−1^ (3.1 (95% Cl 1.6–5.8)) were identified as independent risk factors for ED. The *calculated* probability of ED was 20% (95% Cl 10–31) in patients with both risk factors, 6% (95% Cl 4–9) for patients with only 1 risk factor, and 1.7% (95% Cl 0.9–3) for patients with none of these 2 risk factors. In the CLB-97, CLB-NHL and MBC validation series, the observed incidences of early death in patients with both risk factors were 19%, 25% and 40% respectively and did not differ significantly from those calculated in the model. In conclusion, poor performance status and lymphopenia identify a subgroup of patients at high risk for early death after chemotherapy. © 2001 Cancer Research Campaignhttp://www.bjcancer.com


*British Journal of Cancer* (2001) **85**(6), 816–822
© 2001 Cancer Research Campaign
doi: 10.1054/ bjoc.2001.2011, available online at http://www.idealibrary.com on IDEAL
http://www.bjcancer.com

### Identification of patients at risk for early death after conventional chemotherapy in solid tumours and lymphomas

**I Ray-Coquard[1], H Ghesquière[1], T Bachelot[1], C Borg[1], P Biron[1], C Sebban[1], A LeCesne[2], F Chauvin[1] and J-Y Blay[1,3] for the ELYPSE Study Group**

[1]Centre Léon Bérard, Lyon, France; [2]Institut Gustave Roussy, Paris, France; [3]Hospital Edouard Herriot, Lyon, France

**Summary** 1–5% of cancer patients treated with cytotoxic chemotherapy die within a month after the administration of chemotherapy. Risk factors for these early deaths (ED) are not well known. The purpose of this study was to establish a risk model for ED after chemotherapy applicable to all tumour types. The model was delineated in a series of 1051 cancer patients receiving a first course of chemotherapy in the Department of Medicine of the Centre Léon Bérard (CLB) in 1996 (CLB-1996 cohort), and then validated in a series of patients treated in the same department in 1997 (CLB-1997), in a prospective cohort of patients with aggressive non-Hodgkin's lymphoma (NHL) (CLB-NHL), and in a prospective cohort of patients with metastatic breast cancer (MBC series) receiving first-line chemotherapy. In the CLB-1996 series, 43 patients (4.1%) experienced early. In univariate analysis, age > 60, PS > 1, lymphocyte (ly) count ≤ 700 $\mu l^{-1}$ immediately prior to chemotherapy (d1), d1-platelet count ≤ 150 $Gl^{-1}$, and the type of chemotherapy were significantly correlated to the risk of early death ($P \leq 0.01$). Using logistic regression, PS > 1 (hazard ratio 3.9 (95% Cl 2.0–7.5)) and d1-ly count ≤ 700 $\mu l^{-1}$ (3.1 (95% Cl 1.6–5.8)) were identified as independent risk factors for ED. The *calculated* probability of ED was 20% (95% Cl 10–31) in patients with both risk factors, 6% (95% Cl 4–9) for patients with only 1 risk factor, and 1.7% (95% Cl 0.9–3) for patients with none of these 2 risk factors. In the CLB-97, CLB-NHL and MBC validation series, the observed incidences of early death in patients with both risk factors were 19%, 25% and 40% respectively and did not differ significantly from those calculated in the model. In conclusion, poor performance status and lymphopenia identify a subgroup of patients at high risk for early death after chemotherapy. © 2001 Cancer Research Campaign http://www.bjcancer.com

**Keywords**: lymphopenia; treatment-related death; cancer; risk factors; chemotherapy; cancer; palliative

Death within a month after the administration of cancer chemotherapy is a rare event occurring after 0.5% to 5% of conventional chemotherapy courses (Sculier et al, 1984; Antman et al, 1993; Fisher et al, 1993; Hussain et al, 1991; Radford et al, 1993; Lilienbaum et al, 1998). These early deaths may result either from the toxicity of chemotherapy, disease progression or both. Sepsis-related death in neutropenic patients is the most frequent cause of mortality due to the toxicity of chemotherapy, although other causes such as renal, cardiac or neurological lethal toxicities are not infrequent (Sculier et al, 1984; Hussain et al, 1993; Radford et al, 1996; Lilienbaum et al, 1998). Parameters influencing the risk of early death after chemotherapy in cancer patients are not well known, although it is generally reported that a poor performance status predicts a poor outcome after chemotherapy in most cancer types (Morittu et al, 1989; Komaki et al, 1993; Shipp et al, 1993; Gomez et al, 1998). This work was initiated to identify risk factors for early death in patients receiving chemotherapy. Early death was defined here as a death within 31 days after the administration of chemotherapy, an event for which a contribution of chemotherapy is generally considered in clinical studies (Kramer et al, 1979; Begaud et al, 1985). A risk model based on patient's characteristics was delineated and then validated in 3 distinct series of patients.

*Received 6 November 2000*
*Revised 7 June 2001*
*Accepted 3 July 2001*

*Correspondence to:* J-Y Blay

### PATIENTS AND METHODS

The method used in this study was (1) to identify independent risk factors for early death in a large retrospective series of patients with various tumour types, (2) to delineate a risk index for early death and (3) to validate this index in 3 different series of patients.

### Criteria for patient selection

4 distinct cohorts of patients are considered in this analysis. In all 4 series, the selection criteria for patients were identical and as follows: patients had to be older than 17 years and to be treated with a chemotherapy regimen. Patients previously treated outside the centre were eligible. Patients receiving chemotherapy regimens administered daily, or treated concomitantly with interferon or interleukin-2 were excluded as well as patients receiving high-dose chemotherapy regimens requiring bone marrow or peripheral blood stem cell reinjection. Since baseline blood cell counts were tested as prognostic factors in view of their prognostic value for haematological toxicity, leukaemia and follicular lymphomas with blood involvement were excluded from this study: these represented 7 patients in the CLB-1996 series, of whom none had lymphocyte count under 700 $\mu l^{-1}$ and none died within 31 days after the first chemotherapy administration. Each patient was included only once in the 4 series.

The retrospective group of 1996 (CLB-1996) comprised all patients treated with cytotoxic chemotherapy in the Department of

Medicine of the Centre Léon Bérard in 1996 who matched the selection criteria. 3223 chemotherapy courses were given to 1116 patients in 1996. Every patient was analysed only for his/her first course of chemotherapy given in 1996 in the Centre Léon Bérard. Information about histology, stage at diagnosis, line of chemotherapy, number of previous courses, primary tumour site, chemotherapy regimen, gender, age, performance status (PS), blood cell count on day 1 (d1) just before the administration of chemotherapy were collected. Patients with a missing blood cell count at day 1 ($n = 65$) were excluded. Therefore 1051 of the 1116 patients (94%) were analysed. Co-morbidities susceptible to increase the risk of death were reported in 178 of these 1051 patients (17%): 99 (9%) had cardiac diseases, 23 (2%) hepatic diseases, 47 (5%) lung diseases, 9 (1%) neuropsychiatric disorders. The risk model was established in this retrospective series.

The first validation group (CLB-1997) is the retrospective series of 797 patients treated in the Centre Léon Bérard during 1997 *and* who had not received chemotherapy in the Department of Medicine of the Centre Léon Bérard in 1996. All patients were analysed only for their first course of chemotherapy given in 1997 in the Centre Léon Bérard.

The second validation group (NHL-CLB) is a cohort of 149 non-pretreated patients with intermediate- or high-grade non-Hodgkin's lymphoma who received a first course of conventional chemotherapy regimen in the Centre Léon Berard between 1987 and 1995 while included in prospective multicentric phase III trials of the multicentric groupe d'étude des lymphomes de l'adulte (GELA) group. There were no missing data in this series and all patients were evaluated.

The third validation group (MBC) is a multicentric prospective phase III series including 309 female patients with hormono-resistant metastatic breast cancer treated with the CEF regimen (cyclophosphamide, epirubicin, fluorouracile) in first line in both the Centre Léon Bérard ($n = 197$, 64%) and in general hospitals of the French Rhône-Alpes region ($n = 112$, 36%) between 1985 and 1990 (Chauvin et al, 1990). Only 286 of the 309 (93%) patients included in this series were analysed because of missing day-1 blood cell count ($n = 5$) or missing performance status ($n = 18$).

The main characteristics of the 4 series of patients are presented in the Table 1.

### Definition of early death

The major endpoint of this study was the death of the patient within 31 days following day 1 of chemotherapy administration, referred to as early death, resulting either from toxicity, disease progression or both. In most clinical trials in oncology, and according to Good Clinical Practice, death within 31 days is an event for which a possible role of chemotherapy is generally considered since chronological delay is suggestive (Kramer et al, 1979; Begaud et al, 1985).

### Chemotherapy regimens

Chemotherapy regimens were separated into 2 subgroups according to the doses of drugs administered, as reported in previous ELYPSE studies (Blay et al, 1996, 1997, 1998; Ray-Coquard et al, 1999). The 'high-risk regimen' were defined according to the initial recommendations of the French Ministry of Health for the use of G-CSF or GM-CSF: in these recommendations, the use of G-CSF or GM-CSF was restricted to those patients receiving regimens including at least these doses of chemotherapy (for anthracyclines, alkylating agents and VP16). For CDDP and cytosine arabinoside, the threshold of doses chosen were those used internally within the CLB to select patients for primary prophylaxis with nG-CSF or GM-CSF. 'High risk' chemotherapy regimens, as defined in this study are regimens containing doxorubicin or epirubicin $\geq 90$ mg $m^{-2}$, or cisplatin $\geq 100$ mg $m^{-2}$, or ifosfamide $\geq 9$ g $m^{-2}$, or cyclophosphamide $\geq 1$ g $m^{-2}$, or etoposide $\geq 500$ mg $m^{-2}$, or cytarabine $\geq 1$ g $m^{-2}$ per course. The other subgroup includes all other chemotherapy regimens.

### Statistical analysis

Statistical analysis was performed using the procedures of the SPSS® 8.0 program (Chicago: SPSS, Inc, 1998) following a 3-step design.

**Step 1:** Risk factors for early death were tested in univariate and multivariate analysis in the 1051 patients of the CLB-1996 series. The correlation between a clinical or a biological parameter and the incidence of early death was performed using the Pearson $\chi^2$ test or Fisher's exact test. A logistic regression including the parameters studied in the univariate analysis was performed using the logistic program of SPSS®: a forward regression procedure was used with a $P$ value $< 0.05$ for entry. Risk factors (e.g. PS > 1) and end-point (i.e. early death) were introduced in the model as dummy variables. For most biological values, normal levels were considered as the threshold level, with the exception of lymphopenia $\leq 700$ $\mu l^{-1}$, which was selected because of its predictive value for haematological toxicity of chemotherapy in previous studies of the Elypse group (Blay et al, 1996, 1997, 1998; Ray-Coquard et al, 1999). For other parameters (age, stage, number of previous courses of chemotherapy, number of previous lines of chemotherapy), the most relevant threshold was used in univariate analysis. For instance, we tested different cut-off values for age every 10 years from 30 to 80: 60 was found to be the most relevant threshold in univariate analysis and was kept for this reason.

**Step 2:** Using the parameters value of the logistic regression, 3 subsets of patients were identified according to the cumulative risk of early death, i.e. patients with none, one and both risk factors.

**Step 3:** The discriminant power of this classification was confirmed in the 3 validation series by comparing the observed numbers of early deaths to the calculated number of deaths according to the model using a $\chi^2$ test. Finally, the negative predictive value (NPV) of the model was calculated in CLB-1997, CLB-NHL and MBC series as the posterior probability of no early death for patients without risk factors (1-NPV is the probability of early death for patients without risk factors). The relative risk of the high-risk group is estimated as the positive predictive value divided by 1-NPV.

In addition, the incidence of death at 3 months was investigated in the 3 risk groups of the 4 series.

### RESULTS

### Risk factors for early death after chemotherapy

43 of the 1051 patients of the CLB-1996 group (4.1%) died within the 31 days following the administration of their first course of cytotoxic chemotherapy in 1996 (Table 2). In 23 cases (53%) death was considered to result only from the toxicity of the treatment (18 septic shock, 2 cardiac failures, 2 pulmonary embolism

*© 2001 Cancer Research Campaign*

**Table 1** Characteristics of the patients

| | CLB-1996 (*n* = 1051) | | CLB-1997 (*n* = 797) | | CLB LNH (*n* = 149) | | MBC (*n* = 286) | | TOTAL (*n* = 2283) | |
|---|---|---|---|---|---|---|---|---|---|---|
| | No of pts | (%) | No of pts | (%) | No of pts | (%) | No of pts | (%) | No of pts | (%) |
| Median age (range) | 55 (18–86) | | 55 (18–88) | | 70 (20–93) | | 56 (27–70) | | 57 (18–93) | |
| Age, years | | | | | | | | | | |
| < 60 | 655 | 62 | 489 | 61 | 37 | 25 | 193 | 67 | 1374 | 60 |
| ≥ 60 | 396 | 38 | 308 | 39 | 112 | 75 | 93 | 33 | 909 | 40 |
| Sex | | | | | | | | | | |
| Female | 567 | 54 | 442 | 55 | 68 | 46 | 286 | 100 | 1363 | 60 |
| Male | 484 | 46 | 355 | 45 | 81 | 54 | 0 | 0 | 920 | 40 |
| Diagnosis | | | | | | | | | | |
| Carcinoma | | | | | | | | | | |
| Breast | 247 | 24 | 223 | 28 | 0 | 0 | 286 | 100 | 756 | 33 |
| Colon-rectum | 96 | 9 | 57 | 7 | 0 | 0 | 0 | 0 | 153 | 8 |
| Ovary | 90 | 9 | 41 | 5 | 0 | 0 | 0 | 0 | 131 | 7 |
| Head and neck | 103 | 10 | 54 | 7 | 0 | 0 | 0 | 0 | 157 | 8 |
| Other gastrointestinal | 92 | 9 | 39 | 5 | 0 | 0 | 0 | 0 | 131 | 7 |
| Lung cancer | 61 | 6 | 67 | 8 | 0 | 0 | 0 | 0 | 128 | 6 |
| Other carcinoma (genitourinary, gynaecologic, thyroid, unknown) | 97 | 9 | 103 | 13 | 0 | 0 | 0 | 0 | 200 | 9 |
| Bane and soft tissue sarcoma | 68 | 6 | 53 | 7 | 0 | 0 | 0 | 0 | 121 | 6 |
| Lymphoma | 93 | 9 | 61 | 8 | 149 | 100 | 0 | 0 | 303 | 13 |
| Other[a] | 104 | 10 | 99 | 12 | 0 | 0 | 0 | 0 | 203 | 9 |
| High-risk chemotherapy[b] | | | | | | | | | | |
| Yes | 103 | 10 | 199 | 25 | 74 | 50 | 0 | 0 | 376 | 17 |
| No | 948 | 90 | 598 | 75 | 75 | 50 | 286 | 100 | 1907 | 83 |
| Chemotherapy regimens | | | | | | | | | | |
| ACVBP/ECVBP | 14 | 1 | 18 | 3 | 74 | 50 | 0 | 0 | 106 | 5 |
| Doxorubicin alone | 27 | 3 | 26 | 3 | 0 | 0 | 0 | 0 | 53 | 2 |
| Cisplatin-5FU | 85 | 8 | 33 | 4 | 0 | 0 | 0 | 0 | 118 | 2 |
| Cisplatin-vinorelbine/cisplatin-VP16 | 55 | 5 | 40 | 5 | 0 | 0 | 0 | 0 | 95 | 4 |
| DHAP | 24 | 2 | 16 | 2 | 0 | 0 | 0 | 0 | 40 | 1 |
| CAF/CEF/AC | 115 | 11 | 103 | 13 | 0 | 0 | 286 | 100 | 504 | 22 |
| 5FU-leucoverin/LV5FU2 | 71 | 7 | 37 | 5 | 0 | 0 | 0 | 0 | 108 | 5 |
| MBACOD | 0 | 0 | 0 | 0 | 15 | 10 | 0 | 0 | 15 | 1 |
| MAID | 11 | 1 | 9 | 1 | 0 | 0 | 0 | 0 | 20 | 1 |
| TAXANES | 124 | 12 | 89 | 11 | 0 | 0 | 0 | 0 | 213 | 10 |
| CHOP/CEOP | 25 | 2 | 27 | 3 | 53 | 36 | 0 | 0 | 105 | 5 |
| BEP/EP | 30 | 3 | 31 | 4 | 0 | 0 | 0 | 0 | 61 | 3 |
| Vinorelbine alone | 74 | 7 | 57 | 7 | 0 | 0 | 0 | 0 | 131 | 5 |
| 5FU-leucoverin-oxaliplatin | 34 | 3 | 14 | 2 | 0 | 0 | 0 | 0 | 48 | 2 |
| Other | 362 | 34 | 297 | 37 | 7 | 5 | 0 | 0 | 666 | 29 |
| No of chemotherapy courses | | | | | | | | | | |
| 1 | 822 | 78 | 721 | 90 | 149 | 100 | 286 | 100 | 1978 | 87 |
| ≥ 2 | 229 | 22 | 76 | 10 | 0 | 0 | 0 | 0 | 305 | 13 |
| No of previous lines of chemotherapy | | | | | | | | | | |
| 0 | 618 | 59 | 497 | 62 | 149 | 100 | 127 | 43 | 1391 | 61 |
| ≥ 1 | 433 | 41 | 300 | 38 | 0 | 0 | 159 | 54 | 892 | 39 |
| Stage at diagnostic | | | | | | | | | | |
| 1–2 | 658 | 63 | 510[c] | 64 | 71 | 48 | 143[d] | 55 | 1382 | 61 |
| 3–4 | 393 | 37 | 283[c] | 36 | 78 | 52 | 68[d] | 26 | 822 | 36 |
| Performance status | | | | | | | | | | |
| 0–1 | 881 | 84 | 669 | 84 | 103 | 69 | 172 | 60 | 1825 | 80 |
| > 1 | 170 | 16 | 128 | 16 | 46 | 31 | 114 | 40 | 458 | 20 |

[a]Melanoma, germ cell tumours, myeloma, mesothelioma, glioma, [b]see Material and methods, [c]missing data = 4, [d]missing data: *n* = 5.

in patients bedridden following chemotherapy, 1 suicide in the context of severe mucositis, marrow aplasia and depression following administration of a 5FU-CDDP-corticosteroids regimen), while in 20 cases (47%) death was considered to be the consequence of progressive disease with a possible contribution of the toxicity of chemotherapy. In univariate analysis, age over 60 years, the type of chemotherapy, performance status (PS) > 1, platelet count < 150 000 $\mu l^{-1}$ and lymphocyte count ≤ 700 $\mu l^{-1}$ immediately prior to the initiation of chemotherapy (d1) were found to be significantly correlated ($P < 0.05$) to the risk of early death (Table 3). In contrast, stage at diagnosis, gender, d1 polymorphonuclear leukocyte (PMN) count < 1500 $\mu l^{-1}$, d1 haemoglobin count < 12 g $dl^{-1}$, the line of chemotherapy regimen (first vs. other), chemotherapy regimen containing cisplatin, single agent vs. combination chemotherapy, and the presence of co-morbidities were not significantly correlated with the incidence of

© 2001 Cancer Research Campaign

**Table 2** Description of regimens given in patients experiencing early death

| Regimen | Total *n* | Death *n* (%) | Day of death median (range) |
|---|---|---|---|
| Vinorelbine weekly | 74 | 7 (9%) | 18 (9–27) |
| Cisplatin-5 fluorouracil | 85 | 5 (6%) | 18 (5–31) |
| Vinorelbine-cisplatin | 34 | 4 (12%) | 16 (7–28) |
| Cisplatin-etoposide | 21 | 3 (14%) | 14 (10–23) |
| CAF/CEF | 115 | 3 (3%) | 9 (3–15) |
| Other platinum-containing regimens | 52 | 6 (12%) | 9 (2–24) |
| CHOP-like regimens | 29 | 3 (10%) | 16 (1–17) |
| Mitoxantrone-containing regimens | 13 | 3 (23%) | 11 (5–17) |
| Ifosfamide-containing regimens | 30 | 3 (10%) | 20 (18–23) |
| 5-FU-containing regimens | 80 | 2 (2%) | 14 (10–18) |
| Other | 37 | 4 (11%) | 7 (4–23) |

early death (Table 3). Interestingly, although the number of previous courses of chemotherapy was significantly correlated to lymphopenia counts (median lymphocyte count 1330 vs 1190 $\mu l^{-1}$ in patients who previously received 0–1 vs >1 course, $P = 0.007$), this parameter was not significantly correlated to the risk of ED in this series. A logistic regression was performed to identify independent risk factors for early death including the parameters tested in the univariate analysis. The parameters identified as independent risk factors for early death were PS > 1 (OR: 3.9 (95% CI 2–7.5)), and d1-lymphocyte count ≤ 700 $\mu l^{-1}$ (OR: 3.1 (95% CI 1.8–5.8)). Of note, the same 2 independent parameters influenced the risk of ED due to toxicity only in this series (23 patients).

### A risk model for early death after chemotherapy

Since the relative risk associated with the 2 independent factors were similar, 3 subgroups of patients with a different risk of early death were considered: patients with both risk factors (high-risk group), patients with only 1 of the 2 risk factors (intermediate risk group), patients with none of these 2 risk factors (low-risk group) (Table 4). The calculated probability to die within the 31 days following chemotherapy was 20% (95% CI, 10–31%) for patients of the high-risk group, 6% (95% CI, 4–9%) for patients in the intermediate risk group, 1.7% (95% CI, 0.9–3%) for patients in the low-risk group. The observed incidences of early death in the 3 groups were found to be significantly different in the CLB-1996 cohort ($P < 10^{-5}$). The relative risk of early death (see Statistical methods) in the high-risk group is 7.32 compared to the intermediate- plus low-risk groups.

### Validation of the model (Table 4)

The observed incidence of early death in the 4 series were significantly different (43 of 1051 (4.1%), vs. 37 of 797 (4.6%), vs. 15 of 149 (10%), vs. 11 of 286 (3.8%) ($\chi^2 = 11.1$, $P = 0.01$) in the CLB-1996, in the CLB-1997, in the CLB-NHL and in the MBC series, respectively. In the CLB-1997 series, the observed incidence of early death was 19% (8 of 42), 9% (20 of 218), 1.7% (9 of 537) respectively in the high-, intermediate and low-risk groups ($P < 10^{-5}$) (Table 4). In the CLB-NHL series, the observed incidence of early death was 40% (4 of 10), 9% (5 of 53), 7% (6 of 86) respectively in these 3 groups ($P < 10^{-5}$) (Table 4). In the MBC series, 4 of 16 (25%) patients with both risk factors experienced early death as compared to 5 of 126 (4%) for the intermediate-risk group and 2 of 144 (2%) for the low-risk group ($P < 10^{-3}$). The negative predictive value of the model is respectively 0.96, 0.92 and 0.97 for the 3 validation series as compared to 0.97 in the CLB-1996 series. The relative risks of early death of the high-risk group vs. intermediate- and low-risk group in the 3 validation series are, respectively, 4.96, 5.05 and 9.64. The calculated number of deaths according to the model was not significantly different from the observed number of death in the 3 validation series (not shown).

### Survival beyond 1 month in the 3 groups

The observed incidence of death at 3 months for patients of the high- intermediate and low-risk groups were 45% (29 of 65), 18% (49 of 274) and 4.8% (34 of 712) in the CLB-1996 cohort, 43% (18 of 42), 22% (49 of 218) and 5% (89 of 537) in the CLB-1997 cohort, 50% (5 of 10), 26% (14 of 53) and 13% (11 of 86) in CLB-NHL cohort, 38% (6 of 16), 16% (20 of 126) and 3% (4 of 144) in the MBC series. In these 4 series, 44% (58/133) of the patients of the high-risk group therefore died within 3 months.

Finally, it was asked whether the present model could identify patients at high risk for early death *at any given point* during their career receiving chemotherapy. Among the database of 3223 chemotherapy courses given in the Department of Medicine of the CLB in 1996, 2481 courses were given in patients in whom both day-1 PS and day-1 lymphocyte counts were available. Overall, 78 patients experienced early death following one of these 2478 assessable courses in 1996: 25 ED occurred after 154 (16%) courses given to patients in the high-risk group, 35 after 788 courses (4%) given to patients in the intermediate-risk group, and 18 after 1539 courses (1.2%) to patients in the low-risk group ($\chi^2 = 110$, $P < 10^{-5}$).

### DISCUSSION

Although rare, early death after the administration of chemotherapy is a major concern for the physician prescribing chemotherapy. The identification of patients at high risk for this event would be useful in clinical practice in order to propose an intensive surveillance between courses, or alternatively to make the decision to postpone the administration of chemotherapy, in particular in palliative situations. There is however, no published model enabling the identification of patients at high risk for early death following chemotherapy. The objective of this study was to analyse risk factors for early mortality after the administration of conventional chemotherapy, and to delineate a simple risk index for early death after chemotherapy which could be used in all tumour types in clinical practice. Only simple and readily available clinical and biological parameters in routine clinical practice were considered in this study.

*© 2001 Cancer Research Campaign* 

**Table 3** Risk factors for early death after chemotherapy in the CLB 1996 series

| | Number of patients (%) | Early Death | | *P* |
|---|---|---|---|---|
| | | *n* | % | |
| Age, years | | | | 0.04 |
| ≤ 60 | 655 (62) | 21 | 3.2 | |
| > 60 | 396 (38) | 22 | 5.6 | |
| Stage at diagnostic | | | | 0.32 |
| 1–2 | 658 (63) | 25 | 3.8 | |
| 3–4 | 393 (37) | 18 | 4.6 | |
| Sex | | | | 0.41 |
| Female | 567 (54) | 22 | 3.9 | |
| Male | 484 (46) | 21 | 4.3 | |
| # of chemotherapy courses | | | | 0.13 |
| 1 | 822 (78) | 37 | 4.5 | |
| ≥ 2 | 229 (22) | 6 | 2.6 | |
| # of previous lines of chemotherapy | | | | 0.19 |
| 0 | 618 (59) | 22 | 3.6 | |
| ≥ 1 | 433 (41) | 21 | 4.8 | |
| High risk chemotherapy[a] | | | | 0.01 |
| Yes | 103 (10) | 0 | 0 | |
| No | 948 (90) | 43 | 4.5 | |
| Co-morbidities | | | | 0.29 |
| Yes | 178 (17) | 9 | 5 | |
| No | 873 (83) | 34 | 4 | |
| Histological type | | | | 0.92 |
| Carcinoma | 786 (75) | 33 | 4.2 | |
| Lymphoma | 93 (9) | 4 | 3.5 | |
| Sarcoma | 68 (6) | 3 | 4.3 | |
| Other[b] | 104 (10) | 3 | 3.5 | |
| Performance status | | | | <0.001 |
| 0–1 | 881 (84) | 23 | 2.6 | |
| > 1 | 170 (16) | 20 | 11.8 | |
| d1 hemoglobin count | | | | 0.07 |
| < 12 g dl–1 | 510 (48) | 26 | 5.1 | |
| ≥ 12 g dl–1 | 541 (52) | 17 | 3.1 | |
| d1 lymphocyte count | | | | <0.001 |
| ≤ 700 µl–1 | 234 (22) | 22 | 9.4 | |
| > 700 µl–1 | 817 (78) | 21 | 2.6 | |
| d1 platelet count | | | | 0.01 |
| < 150 000 µl–1 | 100 (10) | 9 | 9 | |
| ≥ 150 000 µl–1 | 951 (90) | 34 | 3.6 | |
| d1 PMN count | | | | 0.7 |
| < 1500 $\mu l^{-1}$ | 26 (3) | 1 | 3.8 | |
| ≥ 1500 $\mu l^{-1}$ | 1025 (97) | 42 | 4.1 | |
| Regimen containing platinum salts | | | | 0.5 |
| Yes | 430 (41) | 18 | 4.2 | |
| No | 621 (59) | 25 | 4 | |
| Single agent chemotherapy | | | | 0.17 |
| Yes | 288 (27) | 15 | 5.2 | |
| No | 763 (73) | 28 | 3.7 | |
| Prophylactic G-CSF | | | | 0.6 |
| Yes | 101 (10) | 4 | 4 | |
| No | 950 (90) | 39 | 4.1 | |

[a]See Materials and Methods; [b]see Table 1.

The first difficulty in addressing this issue is the definition of early death and the contribution of chemotherapy to this outcome. In most toxicity scale grading published to date, and in particular for the analysis of clinical trials, it is considered that death within a month following the administration of chemotherapy should be attributed, unless otherwise proven, to the toxicity of chemotherapy (Kramer et al, 1979; Begaud et al, 1985). However, the causality relationship between administration of chemotherapy and the death of the patient is sometimes difficult to demonstrate. For practical purpose, the major endpoint of this study was chosen to be ‘early death’, as defined by death within 31 days following administration of chemotherapy: in the present study, early death could result from both the toxicity of chemotherapy and/or from chemotherapy failure, i.e. disease progression.

It has been reported that poor performance status, poor bone marrow reserve, pre-existing organ dysfunction and denutrition are risk factors for chemotherapy-related mortality (Morritu et al, 1989; Komaki et al, 1993; Kimmick et al, 1997). In the present

 © 2001 Cancer Research Campaign

**Table 4** Incidence of early death in the 3 groups according to the risk model

| | | Observed incidence | | | | | | | | | |
|---|---|---|---|---|---|---|---|---|---|---|---|
| | | | | Validation series | | | | | | | |
| | | CLB-1996 | | CLB-1997 | | CLB-NHL | | MBC | | Total | |
| | Calculated Incidence (95% CI) | *n* | % | *n* | % | *n* | % | *n* | % | *n* | % |
| 2 risk factors | 20% (10–31) | 14/65 | 21.5% | 8/42 | 19.0% | 4/10 | 40% | 4/16 | 25% | 30/133 | 22.5% |
| 1 risk factor | 6% (4–9) | 14/274 | 5.1% | 20/218 | 9.1% | 5/53 | 9.4% | 5/126 | 3.9% | 44/671 | 6.5% |
| no risk factor | 1.7% (0.9–3) | 15/712 | 2.1% | 9/537 | 1.6% | 6/86 | 6.9% | 2/144 | 1.4% | 32/1479 | 2.1% |

The differences between the observed numbers and calculated numbers (i.e. total number of patients × calculated incidences) of early death were not significantly different in the 3 risk groups of the 4 validation series ($\chi^2$ = 0.05, *P* = 0.82; and and $\chi^2$ = 0.55, *P* = 0.46 for the CLB-1996 and CLB-1997 series, respectively; $\chi^2$ = 3.14, *P* = 0.08; for the CLB-NHL series, $\chi^2$ = 0.17, *P* = 0.67; for the MBC series).

study, only 2 independent risk factors for early death were identified among those tested: performance status >1 and lymphopenia ≤ 700 $\mu l^{-1}$. Performance status has already been reported as an independent predictor for chemotherapy-related death in lymphomas and lung carcinomas (Morittu et al, 1989; Komaki et al, 1993; Shipp et al, 1993; Gomez et al, 1998) and has also been found to be correlated to the risk of severe infection in cancer patients (Hussain et al, 1991; Kimmick et al, 1997).

Lymphopenia, in contrast, had not been previously reported as a risk factor for early death after chemotherapy in HIV-negative patients, although it is an independent prognostic factor for febrile neutropenia, thrombocytopenia, and anaemia in studies of the ELYPSE group (Blay et al, 1996, 1997, 1988; Ray-Coquard et al, 1999), and a prognostic factor for overall survival in a study published in 1970 (Riesco, 1970). The threshold level of 700 lymphocytes per µl was selected because of its predictive value for haematological toxicities in these previous studies but its biological significance remains unclear (Blay et al, 1996, 1997, 1988; Ray-Coquard et al, 1999). At the present time, it is not known whether lymphopenia is restricted or not to a specific lymphocyte subset in these patients. The mechanism of lymphopenia in cancer patients is unclear. Although it may reflect denutrition in some cases (Marks, 1977; Grant et al, 1981; Hussain et al, 1991), this is probably not the sole cause since other parameters of denutrition, such as weight loss and hypoalbuminaemia are observed in less than 40% of lymphopenic cancer patients (now shown). Of note, since nutritional status was not documented for each patient in the electronic file from which data were extracted, this specific point was not tested as a predictive factor in these series.

Lymphopenia may also result from cumulative myelosuppression from prior chemotherapy courses: indeed a significant correlation was observed between the number of previous courses and lymphopenia. Among possible causes, lymphopenia may result from a destruction of lymphocytes elicited by the tumour and/or from an impairment of the differentiation of lymphocyte progenitors in vivo in cancer patients. For instance, it has been recently reported that lymphocytes of cancer patients undergo activation-induced death in vivo (Saito et al, 2000). The contribution of proapoptotic ligands such as FasL or TNFα has been debated since tumour cells frequently produce or induce the production of these cytokines (Strand et al, 1996; Tanaka et al, 1996; Voorzanger et al, 1996; Restifo, 2000). This issue is currently under investigation prospectively.

Although only 2 independent risk factors for early death were identified in this study, its purpose was to delineate a model with predictive value in all tumour types and stages, with the assumption that such a model would likely be more practical for daily clinical use than a variety of different indexes for different cancer types. The method used favoured, therefore, the identification of parameters with a common prognostic value for early death in different cancer types. It cannot be excluded that other clinical or biological parameters may be correlated to early death in specific tumour types, and may improve the predictive value of the model described here for these specific tumour types. However, the model proved to be predictive in heterogeneous cohorts of patients with different tumour types as well as for 2 homogeneous series of patients with metastatic breast carcinoma receiving first-line chemotherapy and previously untreated aggressive NHL.

Finally, it was chosen to report a model in which variables were dichotomized instead of being used as continuous variables because (1) when age, blood cell count etc. were tested as continuous variables in the multivariate model, the final results, in particular the nature of independent variables (performance status and lymphopenia remaining the independent variables) and relative risk for each patient group were not significantly affected, and (2) the calculation of the individual risk for each patient would be less practical using continuous variables for daily clinical use.

The capacity of the model to identify patients at high risk for early death in most tumour types was confirmed by the analysis of 3 distinct cohorts of patients, including 2 homogeneous groups of patients with untreated aggressive NHL and with metastatic breast carcinoma treated with first-line chemotherapy. This is also supported by the analysis of the incidence of early death in high-risk patients of the CLB-1996 and CLB-1997 series which ranged between 12.5% and 33% in the different tumour types (breast, lung, head and neck, colorectal, genitourinary, sarcomas, lymphomas...) and was 2.4- to 12-fold higher than for the remaining patients with the same tumour (not shown). Finally, no interaction between tumour type (carcinoma vs. lymphomas vs. sarcomas vs. other) and performance status or lymphopenia were detected in the logistic regression procedure, further suggesting that this index has a similar value in the different tumour types.

The risk model described here identifies a small subgroup of patients representing 6% of all patients in the 4 series (133 of 2283) with a high risk of early death after chemotherapy, in whom, an intensive surveillance during the interval could be proposed. Actually, the identification of a patient in the high-risk group may be useful in 2 completely opposite situations: first, for patients treated with a curative intent and/or in first-line treatment, in whom a strict

© 2001 Cancer Research Campaign 

clinical surveillance between courses should be performed. Second, for patients in whom treatment is palliative and who are receiving a second or beyond line of chemotherapy; in these patients, the decision to give chemotherapy should probably be carefully discussed, and if done, with similar precautions as in the first group.

### ACKNOWLEDGEMENTS

We thank Lydie Gandon for her contribution to the construction of the database. This work was partly supported by grants from the Comité Départemental de la Ligue contre le Cancer de la Savoie, du Rhône et de la Saône et Loire, and l'association UTILE.

### REFERENCES

Antman K, Crowley J, Balcerzak SP, Rivkin SE, Weiss GR, Elias A, Natale RB, Cooper RM, Barbolie B and Trump DL (1993) An intergroup phase III randomized study of doxorubicin and dacarbazine with or without ifosfamide and mesna in advanced soft tissue and bone sarcomas. *J Clin Oncol* **11**: 1276–1285

Begaud B, Evreux JC, Jouglard J and Lagier G (1985) Unexpected or toxic drug reaction assessment (imputation). Actualization of the method used in France. *Therapie* **40**: 115–122

Blay JY, Chauvin F, LeCesne A, Anglaret B, Bouhour D, Lasset C, Freyer G, Philip T and Biron P (1996) Early lymphopenia after cytotoxic chemotherapy as a risk factor for febrile neutropenia. *J Clin Oncol* **14**: 636–639

Blay JY, Ray-Coquard I, Mermet J, Maugard-Louboutin C, Ravaud A, Malet M, Cany L, Salles B, Mayeur D, Boutan-Laroze A, Sevin D, Vuvan S, Peaud PY, Balzon JC, Capdeville D, Suchaud JP, Celerier D, Zouai M, Mongodin B, Caudry Y, Chevreau C, Biron P and LeCEsne A (1997) ELYPSE 1: a multicentric prospective study of prognostic factors for febrile neutropenia after cytotoxic chemotherapy in general and cancer hospitals. *Proc Am Soc Clin Oncol* **16**: 56a, 1997 (Abstr)

Blay JY, LeCesne A, Mermet C, Maugard C, Ravaud A, Chevreau C, Sebban C, Guastalla J, Biron P and Ray-Coquard I (1998) A risk model for thrombocytopenia requiring platelet transfusion after cytotoxic chemotherapy. *Blood* **92**: 405–410

Chauvin F, Magnet M, Lasset C, G. Catimel G, Mayer M, Chevarier P, Jacquin JP, Peaud PY and Clavel M (1990) Prognostic factors in the response of a first line chemotherapy in advanced breast cancer. *Bull Cancer* **77**: 941–947

Fisher IR, Gaynor ER, Dahlberg S, Oken MM, Grogan TM, Mize EM, Glick JH, Coltman CA and Miller TP (1993) Comparison of a standard regimen (CHOP) with three intensive chemotherapy regimens for advanced non-Hodgkin's lymphoma. *N Engl J Med* **328**: 1002–1006

Gomez H, Hidalgo M, Casanova L, Colomer R, Kay Pen DL, Otero J, Rodriguez W, Carracedo C, Cortès-Funes H and Vallejos C (1998) Risk factors for treatment related death in elderly patients with aggressive non-Hodgkin's lymphoma: results of a multivariate analysis. *J Clin Oncol* **16**: 2065–2069

Grant JP, Custer PB and Thurlow J (1981) Current techniques of nutritional assessment. *Surgical Clin North Am* **61**: 437–463

Hussain M, Kish JA, Crane L, Uwayda A, Cummings G, Ensley JF, Tapazoglou E and Al-Sarraf M (1991) The role of infection in the morbidity and mortality of patients with head and neck cancer undergoing multimodality therapy. *Cancer* **67**: 716–721

Kimmick GG, Fleming R, Muss HB and Balducci L (1997) Cancer chemotherapy in older adults. A tolerability perspective. *Drugs Aging* **10**: 34–49

Komaki R, Pajak TF, Byhardt RW, Komaki R, Pajak TF, Byhardt RW, Emami B, Asbell SO, Roach M 3rd, Pedersen JE, Curran WJ Jr, Lattin P and Russell AH (1993) Analysis of early and late deaths on RTOG non-small-cell carcinoma of the lung trials: comparison with CALBGB 8433. *Lung cancer* **10**: 189–197

Kramer MS, Leventhal JM, Hutchinson TA and Feinstein AR (1979) An algorithm for the operational assessment of adverse drug reaction. I. Background, description, and instruction for use. *JAMA* **242**: 623–632

Lilenbaum RC, Langenberg P and Dickersin K (1998) Single agent versus combination chemotherapy in patients with advanced non small cell lung cancer. *Cancer* **82**: 116–126

Marks PN (1977) Nutrition and the cancer problem. *Curr Concepts Nutr* **6**: 7–13

Morittu L, Earl HM, Souhami RL, Ash CM, Tobias JS, Geddes DM, Harper PG and Spiro SG (1989) Patients at high risk of chemotherapy-associated toxicity in small-cell lung cancer. *Br J Cancer* **59**: 801–805.

Radford JA, Ryder WDJ, Dodwell D, Anderson H and Tatcher N (1993) Predicting septic complications of chemotherapy: an analysis of 382 patients treated for small cell lung cancer without dose reduction after major sepsis. *Eur J Cancer* **29**: 81–86

Ray-Coquard I, Le Cesne A, Rubio MT, Mermet J, Maugard C, Ravaud A, Chevreau C, Sebban C, Bachelot T, Biron P and Blay JY (1999) A risk model for severe anemia requiring red blood cell transfusion after cytotoxic conventional chemotherapy regimens. *J Clin Oncol* **17**: 2840–2846

Restifo NP (2000) Not so Fas: Re-evaluating the mechanisms of immune privilege and tumor escape. *Nat Med* **6**: 493–495

Riesco A (1970) Five-year cancer cure: relation to total amount of peripheral lymphocytes and neutrophils. *Cancer* **25**: 135–140

Saito T, Dworacki G, Gooding W, Lotze MT and Whiteside TL (2000) Spontaneous apoptosis of CD8+ T lymphocytes in peripheral blood of patients with advanced melanoma. *Clin Cancer Res* **6**: 1351–1364

Sculier JP, Weerts D and Klastersky J (1984) Causes of deaths in febrile granulocytopenic cancer patients receiving empiric antibody therapy. *Eur J Cancer Clin Oncol*. **20**: 55–60

Shipp MA et al (1993) For the international Non-Hodgkin's lymphoma prognostic factors project. A predictive model for aggressive non-Hodgkin's lymphoma. *N Engl J Med* **329**: 987–994

Strand S, Hofmann WJ, Hugh H, Muller M, Otto G, Strand D, Mariani SM, Stremmel W, Krammer PH and Galle PR (1996) Lymphocyte apoptosis induced by CD95 (APO-1/Fas) ligand expressing tumor cells-a mechanism of immune evasion. *Nat Med* **2**: 1361–1366

Tanaka M, Suda T, Hasa K, Nakamura N, Sato K, Kimura F, Motoyoshi K, Mizuki M, Tagawa S, Ohga S, Hatake K, Drummond AH and Nagata S (1996) Fas ligand in human serum. *Nat Med* **2**: 317–322

Voorzanger N, Touitou R, Garcia E, Delecluse HJ, Rousset F, Joab I, Favrot MC and Blay JY (1996) Interleukin (IL-) 10 and IL-6 are produced in vivo by non Hodgkin's lymphoma cells and act as cooperative growth factors. *Cancer Res* **56**: 5499–5505

 *© 2001 Cancer Research Campaign*
